# Case Report: First report of a novel homozygous nonsense mutation in the *CYBA* gene causing chronic granulomatous disease

**DOI:** 10.3389/fimmu.2026.1744743

**Published:** 2026-03-19

**Authors:** Wen-Yuan Wang, Pan-Pan Ma, Shu-Ying Wang, Yong-Jun Wang

**Affiliations:** 1 Department of Pediatric Respiratory Medicine II, Gansu Provincial Maternal and Child-care Hospital (Gansu Provincial Central Hospital), Lanzhou, Gansu, China; 2 Medical Genetics Center, Gansu Provincial Maternal and Child-care Hospital (Gansu Provincial Central Hospital), Lanzhou, Gansu, China

**Keywords:** bioinformatics analysis, chronic granulomatous disease, CYBA gene, pleural effusion, recurrent severe pneumonia

## Abstract

**Objective:**

To investigate the etiology of recurrent severe pneumonia and pleural effusion in a pediatric patient through pathogenic gene testing and bioinformatics analysis.

**Methods:**

Clinical characteristics and laboratory findings were retrospectively reviewed. Peripheral blood samples were collected from the patient and both parents. Genomic DNA was extracted and subjected to trio whole-exome sequencing (WES) using high-throughput sequencing technology. Sanger sequencing was used to validate suspected pathogenic variants.

**Results:**

Whole-exome sequencing revealed a homozygous variant c.427C>T (p.Q143*) in exon 6 of the *CYBA* gene, inherited from both parents. This variant has not been previously reported in the literature. According to the American College of Medical Genetics and Genomics (ACMG) guidelines, c.427C>T(p.Q143*) was assessed as a likely pathogenic variant (PVS1_Strong+PM2_Supporting+PM3_Supporting). This variant is associated with autosomal recessive chronic granulomatous disease type 4(A22° CGD).

**Conclusion:**

This study identified a novel *CYBA* gene variant in a pediatric patient with recurrent severe pneumonia and pleural effusion, expanding the spectrum of pathogenic variants in this gene and providing evidence for clinical diagnosis and genetic counseling.

## 1 Introduction

Chronic granulomatous disease (CGD) is a rare primary immunodeficiency disorder with significant regional variations in global prevalence. The incidence is approximately 1 in 200,000 to 1 in 250,000 live births in the United States, 1 in 250,000 in Europe, and 1 in 700,000 in Arab countries (1, 2). CGD is characterized by phagocyte dysfunction, leading to an inability to effectively kill ingested microorganisms. Clinically, this manifests as recurrent severe bacterial or fungal infections and the formation of chronic (non-caseating) granulomas due to excessive inflammatory responses (3, 4). Affected organs include the lungs, lymph nodes, liver, skin, and gastrointestinal tract, posing a serious threat to the child’s life (3, 4). In Chinese pediatric cohorts, the lungs are the most commonly affected organ, and approximately 64% of patients exhibit abnormal reactions to BCG vaccination (5). The molecular mechanism of CGD involves defects in genes encoding the phagocyte Nicotinamide Adenine Dinucleotide Phosphate (NADPH) oxidase complex within phagocytes. This results in impaired respiratory burst function, preventing the production of superoxide and thereby failing to effectively eliminate engulfed microorganisms (6, 7). The phagocyte NADPH oxidase complex comprises five subunits encoded by the *CYBB, CYBA, NCF1, NCF2*, and *NCF4* genes. Approximately 70% of cases are caused by mutations in the X-linked recessive *CYBB* gene (encoding NOX2 protein), while the remainder arise from autosomal recessive inheritance (AR), primarily due to mutations in *CYBA, NCF1, NCF2*, and other genes (4, 8, 9). *The CYBA, NCF1*, and *NCF2* genes encode the p22phox, p47phox, and p67phox proteins, respectively. In AR-CGD, mutations in the *CYBA* gene encoding p22phox are relatively rare, accounting for approximately 6% of global cases and classified as AR220CGD (10). The human p22phox gene, called CYBA (OMIM number 233690), is located on the long arm of chromosome 16 at position 24 (16q24: 88,643,288 to 88,651,084, OMIM 608508), and contains six exons and spans 8.5 kb. P22phox is a transmembrane protein that contains 195 amino acids, serving as a core component of the phagocyte NADPH oxidase complex with four N-terminal transmembrane domains and a conserved C-terminal cytoplasmic proline-rich region (6, 11). Functionally, p22phox forms the heterodimeric catalytic core flavocytochrome b558 with gp91phox (also named NOX2, encoded by CYBB), and its C-terminal proline-rich region mediates critical binding to the SH3 domain of p47phox; this interaction is essential for the assembly and activation of the NADPH oxidase complex, as well as the subsequent production of superoxide anions for microbial clearance (6, 11). *NCF1* (encoding p47phox) mutations account for approximately 20% of cases, *NCF2* (encoding p67phox) mutations for about 5%, while *NCF4* mutations are extremely rare (4, 12). Although precise national population-based epidemiological data for CGD incidence in China remain unavailable, recent hospital-based retrospective studies have provided insights into its clinical and genetic features in Chinese pediatric populations. A large single-center study of 159 pediatric CGD patients reported that *CYBB* mutations accounted for 89% of cases, while *CYBA* mutations were identified in only 5% of genetically confirmed patients (5). Individual case reports have further documented the phenotypic diversity of *CYBA*-related CGD in China (13). These findings highlight the rarity of *CYBA* mutations in Chinese CGD patients and underscore the importance of genetic testing in suspected cases.

This paper reports the clinical features, molecular mechanisms, and treatment follow-up results of a pediatric patient with AR-CGD caused by a novel nonsense mutation in the *CYBA* gene. The clinical significance of this variant is discussed in conjunction with a literature review.

## 2 Materials and methods

### 2.1 Clinical data

**Figure 1:**
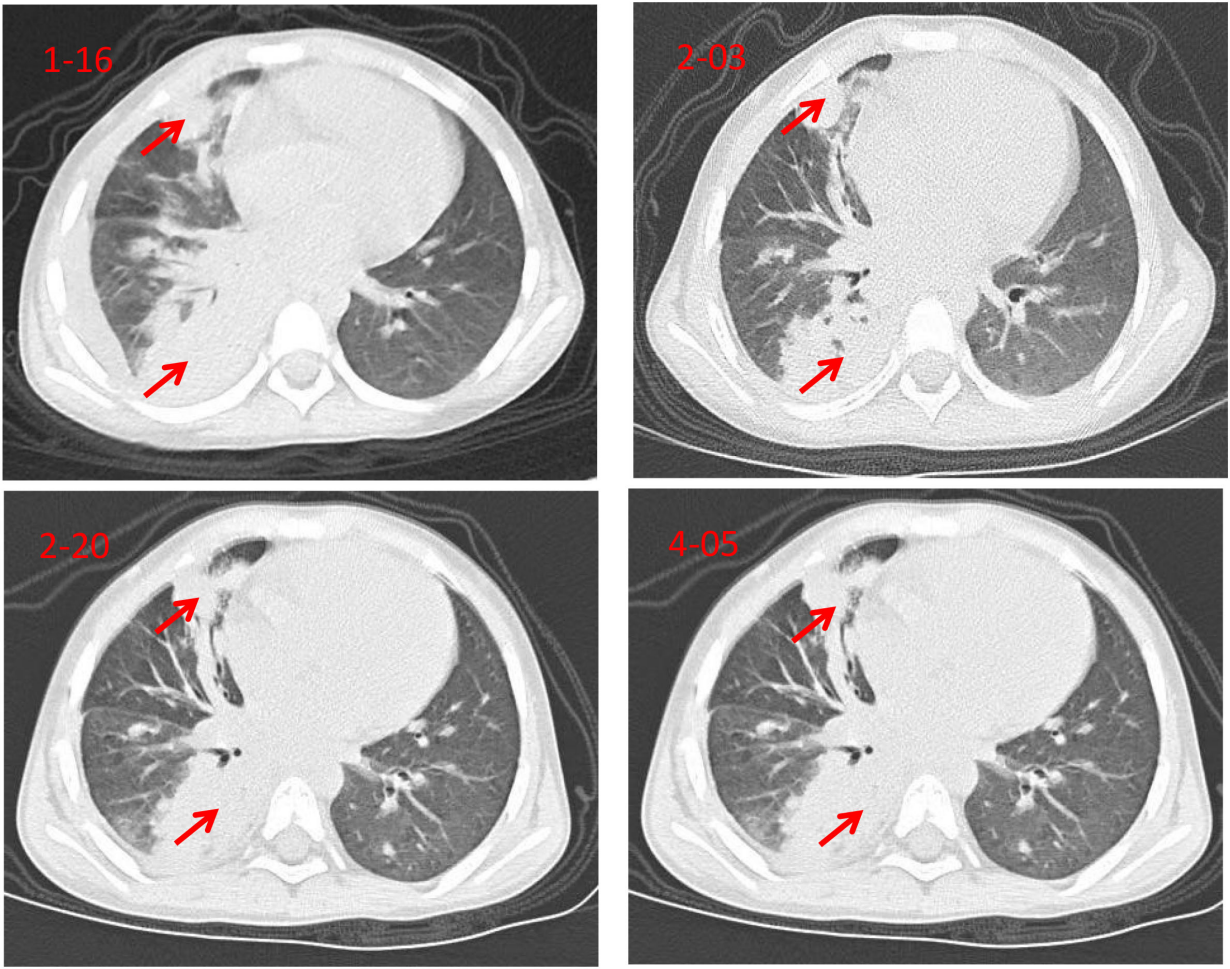
Serial chest CT scans (red arrows indicate bilateral pulmonary consolidation, right pleural effusion, and mediastinal lymphadenopathy; no significant improvement in lesions over time).

The patient was a 5-year-and-1-month-old Han Chinese girl, born to consanguineous parents (first cousins), admitted for “fever for 1 day.” She had a history of three previous hospitalizations for recurrent severe pneumonia and pleural effusion:

First Hospitalization (January 16 - February 3): At 4 years and 9 months old, she was admitted for “fatigue and poor appetite for over 1 month, intermittent fever for over 2 weeks.” Blood tests showed WBC 27.29 × 10^9^/L neutrophils 81.7%, and C-reactive protein (CRP) 207.21 mg/L. Chest CT scans revealed multiple areas of consolidation in both lungs, most prominent in the right lower lobe, with right-sided pleural effusion; follow-up CT showed minimal change (**Figure 1**). *Mycoplasma pneumoniae* IgM was positive (29.63 IU/mL). Bacterial culture of bronchoalveolar lavage fluid (BALF) was negative. Tests and cultures for *Mycoplasma*, *Chlamydia*, and *Tuberculosis* (TB) were negative. She received meropenem and methylprednisolone for 18 days, improving upon discharge.

Second Hospitalization (February 20–March 9): At 4 years and 11 months of age, readmitted for “fever for 6 days.” WBC 19.8 × 10^9^/L neutrophils 78.3%, CRP 156.4 mg/L. Chest CT (February 20): Bilateral pneumonia with multiple areas of consolidation, worsening from previous findings; increased right pleural effusion; Multiple enlarged lymph nodes in the mediastinum and bilateral hilar regions were noted, with the largest measuring approximately 15 mm in short-axis diameter(see **Figure 1**). BALF smear showed Gram-positive bacilli; next-generation sequencing (NGS) detected *Corynebacterium jeikeium*. She received meropenem and azithromycin for 17 days and was discharged stable.

Third Hospitalization (March 23–April 12): Age 5 years old, admitted for “fever for 2 days.” WBC 12.92 × 10^9^/L, neutrophils 72.8%, CRP 149.9 mg/L. Chest CT (April 5) showed bilateral pulmonary consolidation and pleural effusion with minimal change from previous imaging, along with multiple enlarged mediastinal and bilateral hilar lymph nodes (see **Figure 1**). Tuberculous infection T-cell assay, invasive fungal infection testing, and blood cultures were all negative. PPD test was weakly positive; Severe combined immunodeficiency (SCID) screening was normal. To further investigate the underlying cause of recurrent severe infections, a comprehensive immunological and infectious disease workup was performed. Immunophenotyping revealed normal lymphocyte subsets (CD3^+^, CD4^+^, CD8^+^, CD19^+^, CD16/56^+^) and neutrophil counts. Serum immunoglobulin levels (IgG, IgA, IgM, IgE) were within normal ranges for age. Screening for HIV, Epstein-Barr virus, and cytomegalovirus was negative. Peripheral blood smear showed no morphological abnormalities of leukocytes. These results effectively ruled out common primary immunodeficiencies and active viral infections, raising strong clinical suspicion for chronic granulomatous disease (CGD) and prompting genetic testing. Given the recurrent severe pneumonia, whole-exome sequencing (*WES*) was initiated. She received vancomycin and meropenem. In addition, given her history of BCG vaccination, weak PPD positivity, and multiple lymphadenopathies, empiric anti-tuberculosis therapy with isoniazid and rifampicin was initiated. She completed 20 days of treatment and was discharged in stable condition.

The patient had previously received BCG, hepatitis B, measles, and polio vaccinations. She was the second child from the second pregnancy, delivered full-term vaginally. There is no family history of genetic or metabolic diseases. Her 9-year-old brother is healthy.

One day prior to admission (May 16), she developed a high fever (39.9°C without obvious cause, poorly responsive to initial antipyretics. Physical examination revealed pharyngeal congestion, bilateral tonsillar hypertrophy (Grade II) with purulent exudate, and coarse breath sounds. Labs: WBC 13×10^9^/L neutrophils 75.7%, CRP 114.05 mg/L, serum amyloid A > 320.00 mg/L; sputum smear, culture, and acid-fast staining were negative. Considering the history of BCG vaccination, weak PPD positivity, multiple lymphadenopathies, and response to anti-tuberculosis therapy, a clinical diagnosis of disseminated BCG disease was proposed. Due to parental refusal of invasive procedures, alveolar lavage fluid or lymph node *Mycobacterium* culture and *RD1* region gene testing were not performed.

### 2.2 Specimen collection and genomic DNA extraction

Peripheral blood samples were collected from the proband and her parents in accordance with the Declaration of Helsinki. This study was reviewed and approved by our hospital’s Ethics Committee (Approval No: GSFY Review [2025] No. 69). Prior to sample collection, the patient’s parents received pre-test genetic counseling provided by clinical geneticists, including detailed explanations of the purpose of whole-exome sequencing (WES) for diagnosing genetic disorders, potential genetic findings (e.g., pathogenic variants, variants of uncertain significance), and the clinical and familial implications of the results (e.g., genetic counseling for future pregnancies). Written informed consent was obtained from the legal guardians after they fully understood the counseling content.

EDTA-anticoagulated peripheral blood (2–3 mL) was collected from the patient and her parents. Genomic DNA was extracted using the QIAamp DNA Blood Mini Kit (Qiagen, Hilden, Germany) according to the manufacturer’s instructions. DNA concentration (~50 ng/μL) and purity (OD260/280: 1.8-2.0) were measured by NanoDrop 2000 (Thermo Fisher Scientific, Waltham, MA, USA), meeting the quality requirements for high-throughput sequencing.

### 2.3 Trio whole-exome sequencing and bioinformatic analysis

Whole-exome parallel sequencing was performed on the patient and parents using the BGI DNBSEQ-T7 sequencer, achieving an average sequencing depth of 120x All regions attained coverage exceeding 20x to ensure detection accuracy. DNA was submitted for trio whole-exome sequencing (trioWES) to Chigene Co., Ltd. Protein-coding exome enrichment was performed using xGen Exome Research Panel v2.0 (IDT, Iowa, United States) that consists of 429,826 individually synthesized and quality-controlled probes, which targets the 39 Mb protein-coding region (19,396 genes) of the human genome and covers 51 Mb of end-to-end tiled probe space. High-throughput sequencing was performed using a BGI DNBSEQ-T7 sequencer, and not less than 99% of the target sequence was sequenced. The sequencing process was performed by Chigene (Beijing) Translational Medical Research Center Co., Ltd (14).

Raw data were processed using fastp for adapter trimming and low-quality read filtering (Q-score < 20 were removed) to ensure sequencing quality. Paired-end reads were aligned to the Ensembl GRCh37/hg19 reference genome using the Burrows-Wheeler. Aligner (BWA) for primary analysis. Secondary analysis included base quality score recalibration, as well as SNP and short indel calling, which were performed using GATK; high-quality and reliable variants were retained after filtering SNPs and indels based on sequencing depth ((≥10×) and variant quality scores (Q-score ≥30) For tertiary analysis, database-based minor allele frequencies (MAFs) and pathogenicity classification of each identified variant according to ACMG practice guidelines were annotated using an online system independently developed by Chigene (www.chigene.org); no web-based software such as Franklin (https://franklin.genoox.com/) was used in the analysis. This system also integrates multiple software packages for conservation analysis and protein structure prediction. Databases used for MAF annotation included 1000 Genomes, dbSNP, ESP, ExAC, and the Chigene in-house MAF database. Protein structural alterations were predicted using Provean, SIFT, Polyphen2_HDIV, Polyphen2_HVAR, MutationTaster, M-Cap, and REVEL. For supplementary pathogenicity annotation consistent with the ACMG guidelines (15), OMIM, HGMD, and ClinVar databases were used to support variant pathogenicity assessment. To evaluate potential splicing-altering effects of variants, MaxEntScan, dbscSNV, and GTAG tools were employed (14).

### 2.4 Sanger sequencing validation

Primers were designed using the Primer 3.0 online software (http://primer3.ut.ee) (**Table 1**). PCR amplification was performed on the variant site region *of the* candidate pathogenic *gene CYBA*, followed by Sanger sequencing validation. The results were compared with the reference sequence of the *CYBA* gene (NM_000101).

**Table 1:** Sanger sequencing validation primer sequences.

Gene	Chromosomal location	Transcript exon	Nucleotide amino acid	Primer ID	Primer sequence (5'-3')	Fragment length
*CYBA*	chr16:8870 9922	NM_000101;exon6	c.427C>T(p.Q143*)	*CYBA*_E6F	CCTGCGTGGGATTCAGACTTGAGCCT	465bp
				*CYBA*_E6R	AGGCCTCGGGAACCATCGCTACCCCA	

### 2.5 Amino acid variant site conservation analysis

MEGA software was used to perform multi-species sequence conservation analysis of the variant site region. Sequence alignment was conducted using the ClustalW method, generating conservation analysis maps to assess the degree of conservation of the variant site across different species.

### 2.6 Protein tertiary structure prediction

The SWISS-MODEL website (https://swissmodel.expasy.org/) was used to predict the spatial structural models of the wild-type and mutant proteins.

## 3 Results

### 3.1 WES detection results

Analysis of whole-exome sequencing data identified homozygous variants in the *CYBA* gene associated with the patient’s clinical phenotype: c.427C>T(p.Q143*). Concurrently, quality assessment of the sequencing data revealed a Q30 value >90%, average sequencing depth of 120×, target region coverage of 99.8%, and no significant sequencing bias, ensuring the reliability of the detection results.

### 3.2 Sanger sequencing validation of results

**Figure 2:**
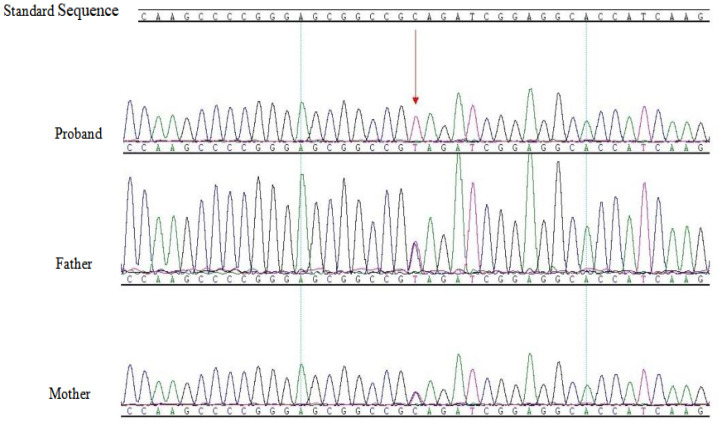
Sanger sequencing validation results of *CYBA* gene variant sites in the patient and her parents:c.427C>T(p.Q143*) mutation diagram, with red arrow indicating the mutation site. The patient carries a homozygous mutation at the c.427C>T(p.Q143*) site, while the father and mother each carry a heterozygous mutation at this site.

Sanger sequencing was applied to validate the whole-exome sequencing results of the patient. Sanger sequencing results were consistent with whole-exome sequencing data, confirming a homozygous c.427C>T (p.Q143*) variant in the *CYBA* gene. Both parents carried a heterozygous variant at this locus (**Figure 2**, red arrow indicates variant site). After querying the HGMD database (HGMD Professional 2024.4), ClinVar database (last accessed February 23, 2026), and PubMed literature, no reports were found for this variant site, confirming it as a novel variant.

### 3.3 Pathogenicity analysis results

In silico pathogenicity analysis of the identified variant was performed using a panel of bioinformatic tools to assess its potential impact on protein structure and function. The c.427C>T (p.Q143*) mutation is a single nucleotide substitution where the 427th nucleotide of the *CYBA* gene mutates from cytosine (C) to thymine (T). This nonsense mutation causes premature termination of translation at the 143rd position of the encoded amino acid sequence.

We reanalyzed the CYBA c.427C>T (p.Q143*) variant using the latest population frequency databases, including the Genome Aggregation Database (gnomAD) v4.1.0 (which integrates ExAC data). All database queries were performed on February 23, 2026. The variant remains absent from ClinVar (last accessed February 23, 2026) and HGMD Professional 2024.4 (HGMD Pro, the latest version available as of the analysis date), confirming its novelty. Based on this reassessment, the ACMG classification remains unchanged.

1. Very strong pathogenic evidence (PVS1_Strong): The *CYBA* c.427C>T (p.Q143*) variant is a nonsense mutation located in exon 6 (the last exon) of *CYBA*, introducing a premature stop codon that truncates 52 amino acids from the C−terminus of the p22phox protein, accounting for 26.7% (143/195) of the total protein length. This truncation eliminates the conserved proline−rich region (PRR, residues 149–162), which is indispensable for binding to the SH3 domain of p47phox and subsequent activation of the NADPH oxidase complex (6, 16). Since the variant occurs in the last exon, it is not predicted to undergo nonsense−mediated decay (NMD). Truncating variants affecting the C−terminal PRR domain of p22phox have been functionally validated to cause complete loss of oxidase activity and are established causes of AR-CGD (6, 10, 17). This variant meets the strict criteria for PVS1_Strong as defined by ACMG/AMP guidelines (15).
2. Supporting pathogenic evidence (PM2_Supporting): Querying the latest Genome Aggregation Database (gnomAD v4.1.0, https://gnomad.broadinstitute.org/) — the primary integrated database for population allele frequency analysis that has incorporated all ExAC data (the ExAC browser is no longer independently available) — the allele frequency of this variant was 0.00008 in the East Asian population and 0 in all other ancestral populations. The overall allele frequency is far below 0.001, which fully meets the PM2_Supporting criteria for autosomal recessive genetic disorders and provides robust population-level evidence for its pathogenicity.
3. Supporting pathogenic evidence (PM3_Supporting): Heterozygosity for this variant was detected in the patient’s parents, who exhibit normal phenotypes, consistent with an autosomal recessive inheritance pattern. The patient is homozygous for the variant, with the genotype showing a strong correlation with the clinical phenotype of AR-CGD. The PM3-Case_Score meets the criterion of 0.5 ≤ PM3-Case_Score < 1.0 for the current case, thus meeting the PM3_Supporting criteria.

No relevant reports exist in literature databases for this variant, and the ClinVar database contains no pathogenicity analysis results for this variant. The reanalysis using updated databases confirmed the initial findings. In summary, this variant is classified as a likely pathogenic variant (PVS1_Strong+PM2_Supporting+PM3_Supporting). The detailed characteristics of the *CYBA* c.427C>T (p.Q143*) variant are summarized in **Table 2**.

**Table 2:** Key information of the *CYBA* c.427C>T (p.Q143*) variant identified in the proband.

Characteristic	Details
Human reference genome	GRCh37/hg19
Cytogenetic location	chr16:88,709,922 (16q24)
Transcript ID	NM_000101
Exon location	Exon 6 (last exon)
Nucleotide variant	c.427C>T (homozygous in proband)
Protein variant	p.Q143* (premature stop codon, 52 C-terminal amino acids lost)
Population frequency	gnomAD v4.1.0: 0.00008 (East Asian population); 0 (other populations)
ACMG classification	Likely Pathogenic (PVS1_Strong+PM2_Supporting+PM3_Supporting)
Inheritance origin	Proband: homozygous; Father: heterozygous; Mother: heterozygous; Elder brother: wild-type (unaffected)
Mutation type	Nonsense mutation (stop-gain variant)

ACMG, American College of Medical Genetics and Genomics; gnomAD, Genome Aggregation Database (ExAC data has been integrated into gnomAD and is no longer independently accessible).

### 3.4 Protein structure prediction and conservation analysis at the variant site

**Figure 3:**
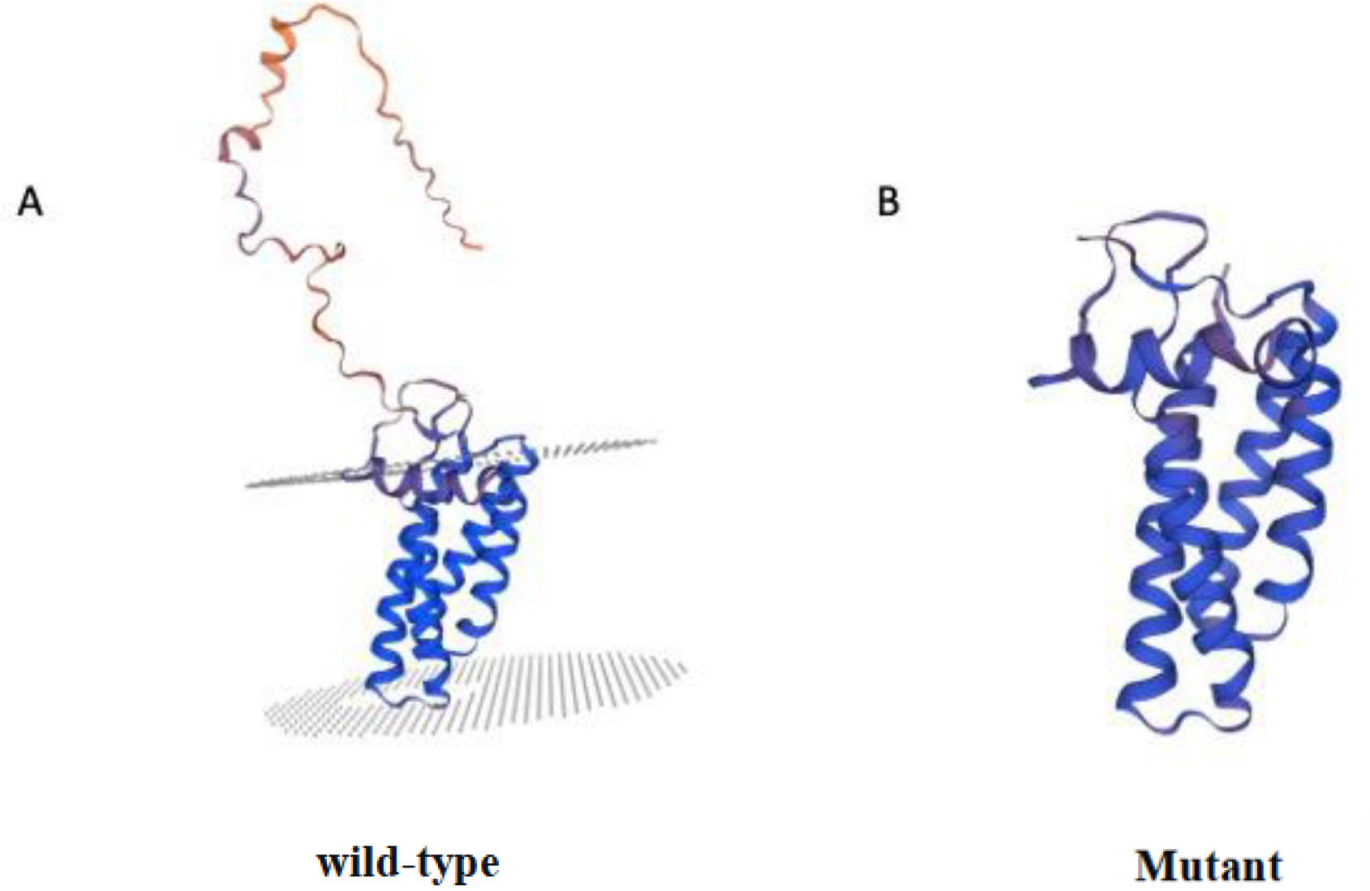
*CYBA* protein tertiary structure model, **(A)***CYBA* protein tertiary structure model; **(B)** Tertiary structure model of the c.427C>T (p.Q143*) mutant site; The c.427C>T (p.Q143*) mutation results in a distinctly truncated *CYBA* protein.

Protein Tertiary Structure Prediction: SWISS-MODEL prediction results indicate that the C-terminal proline-rich region of the wild-type p22phox protein forms a stable α-helical structure, which is the key binding region for the SH3 domain of the p47phox protein (**Figure 3A**). Whereas the c.427C>T (p.Q143*) mutation causes premature termination at position 143, resulting in complete loss of the α-helical structure in the C-terminal proline-rich region and significant alteration of the protein’s spatial conformation (**Figure 3B**). This mutation eliminates the protein’s ability to bind to p47phox.

**Figure 4:**
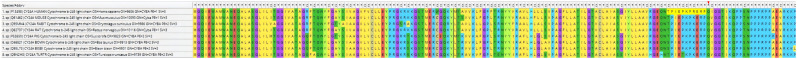
Conservation analysis of the c.427C>T (p.Q143*) mutation site in the *CYBA* gene: Red arrows indicate the amino acid conservation results for the c.427C>T (p.Q143*) mutation site, which is highly conserved across different species.

Conservation analysis: MEGA11 software-based multi-species sequence alignment revealed that the amino acid corresponding to the *CYBA* gene c.427C>T (p.Q143*) mutation site is highly conserved as glutamine (Q) across different species (**Figure 4**, red arrow marks the mutation site), further suggesting that this site is critical for maintaining protein function and that the mutation may lead to protein dysfunction.

## 4 Diagnosis

Autosomal Recessive Chronic Granulomatous Disease (Type 4, A22° CGD), Severe Pneumonia, Pleural Effusion, Disseminated BCG Disease.

## 5 Treatment and follow-up

Initial management included antibiotics (meropenem, azithromycin, vancomycin), IVIG, and nebulization. Empiric anti-tuberculosis therapy (isoniazid, rifampicin) was added due to clinical suspicion of disseminated BCG disease.

Two months post-discharge, at Chongqing Medical University Children’s Hospital, functional tests confirmed CGD: NBT reduction was significantly impaired (unstimulated and LPS-stimulated); DHR assay showed a respiratory burst index of 2.7 (father: 38.3). PPD was ++. Chest CT showed right lower lobe consolidation and lymphadenopathy. Prophylaxis was adjusted to linezolid, isoniazid, ethambutol, itraconazole, and co-trimoxazole. Hematopoietic stem cell transplantation (HSCT) was planned.

**Figure 5:**
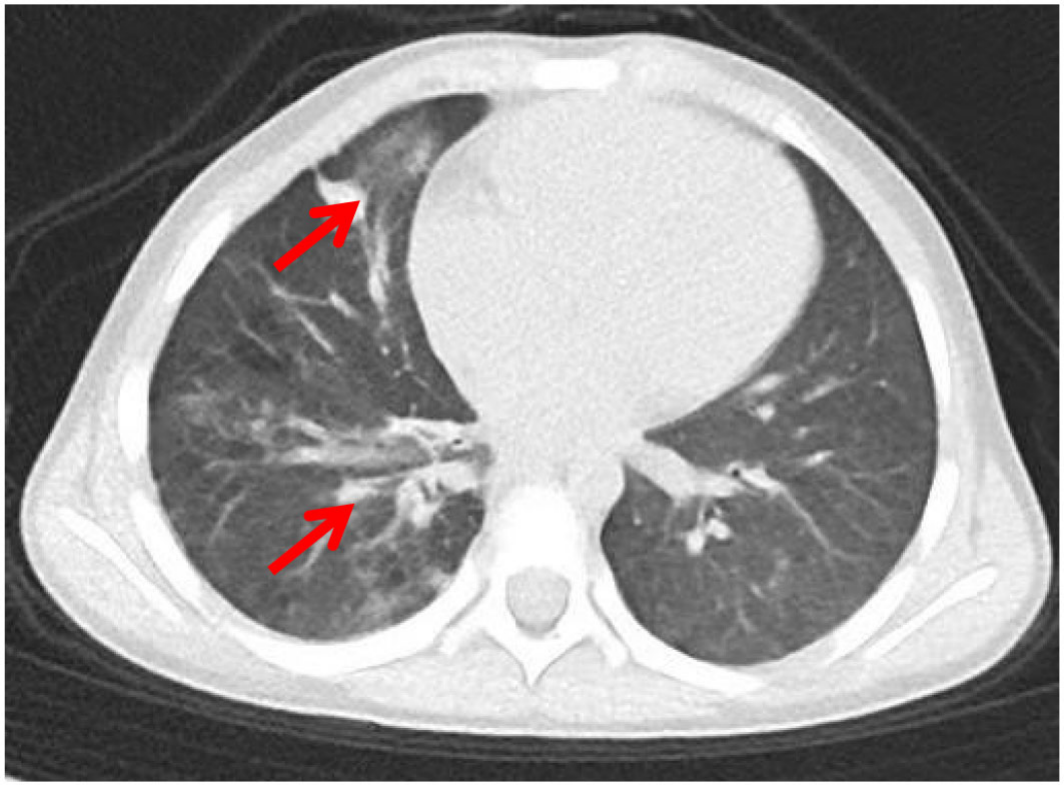
Post-transplant chest CT of the patient (red arrows indicate lesion resolution).

Six months later, the patient remained free of recurrent infections, successfully underwent transplantation with a matched donor, and experienced an uneventful procedure without adverse reactions. One-year post-transplant follow-up revealed no infection recurrence, normal growth and development, and resolution of chest CT lesions (**Figure 5**).

## 6 Discussion

We report a novel homozygous nonsense mutation c.427C>T (p.Q143*) in the *CYBA* gene causing AR-CGD in a child with recurrent severe pneumonia, pleural effusion, and disseminated BCG disease. ACMG criteria classified this previously unreported variant as Likely Pathogenic.

Chronic granulomatous disease (CGD) is characterized by recurrent severe infections beginning in childhood and the formation of chronic granulomas. The lungs are the most commonly affected organ, involved in approximately 80% of cases, and pulmonary complications represent a leading cause of mortality (18). Common pathogens causing pulmonary infections include hydrogen peroxide-positive microorganisms such as *Aspergillus*, *Staphylococcus aureus*, and *Burkholderia cepacia*, along with others like *Serratia marcescens* and *mycobacteria (*5, 19). *Corynebacterium jeikeium*, a normal colonizer of skin and mucous membranes, causes infection only in immunocompromised individuals. This pediatric patient experienced recurrent severe pneumonia with pleural effusion before age 5, with the shortest interval between hospitalizations being only 14 days. *Corynebacterium jeikeium* was detected during the second hospitalization. Imaging consistently showed bilateral pulmonary consolidation and enlarged mediastinal lymph nodes, highly consistent with the clinical phenotype of CGD characterized by “recurrent infections and protracted illness.” Furthermore, this bacterium commonly colonizes human skin (e.g., axillary and inguinal regions) and is classified as an opportunistic pathogen, frequently observed in immunocompromised or chronically hospitalized patients, further suggesting an immunodeficiency. It exhibits intrinsic resistance to multiple antibiotics (e.g., penicillins, cephalosporins, macrolides) but remains susceptible to vancomycin, linezolid, daptomycin. Based on antimicrobial susceptibility testing, intravenous vancomycin therapy was initiated, followed by oral linezolid sequential treatment after discharge. This demonstrates the critical importance of targeted antimicrobial strategies in patients with such unique immunological backgrounds.

In China, where neonatal BCG vaccination is universally implemented, patients with chronic granululomatous disease (CGD) are at significantly elevated risk for BCG-related complications, ranging from localized lymphadenitis to disseminated BCG infection. It is estimated that approximately 75% of mycobacterial infections in CGD patients in this setting are attributable to BCG vaccination (5, 9). Disseminated BCG disease arises from impaired NADPH oxidase activity, which compromises the ability of phagocytes to eliminate BCG bacteria, leading to systemic spread characterized by recurrent fever, lymphadenopathy, pulmonary consolidation, and subsequent granulomatous inflammation (13). In the present case, the patient had a documented BCG vaccination history and exhibited clinical features highly consistent with disseminated BCG disease, which resolved following anti-tuberculosis therapy. Nevertheless, a definitive microbiological diagnosis was limited by the inability to perform Mycobacterium culture or *RD1*-based genetic testing from alveolar lavage or lymph node tissue, as these invasive procedures were declined by the parents. Future management of similar cases should emphasize obtaining appropriate specimens to confirm the etiology wherever feasible.

*CYBA* gene mutations follow an autosomal recessive inheritance pattern and represent one of the extremely rare CGD subtypes clinically. The human p22-phox protein is encoded by the *CYBA* gene (OMIM#233690), located at 16q24.4 (16q24: 88,643,288-88,651,084, OMIM#608508), spans 8.5 kb, and contains six exons (6). Studies confirm p22-phox is a 195-amino acid transmembrane protein, whose encoded amino acids comprise five domains: four N-terminal transmembrane domains and a C-terminal intracellular proline-rich region. The latter domain plays a crucial role in promoting the assembly of the cytoplasmic components of NADPH oxidase and in protein interactions by binding to the SH3 domain of p47-phox (20). Deletions and missense mutations are the primary variant types, while nonsense mutations account for approximately 8.6% of all allele mutations (21).

The *CYBA* gene c.427C>T (p.Q143*) nonsense mutation in this CGD patient is located precisely within this critical region. It causes the 143rd codon to change from glutamine to a stop codon, resulting in the loss of the proline-rich C-terminal domain of the p22-phox protein. This prevents p22-phox from binding to the SH3 domain of p47-phox, and it is hypothesized that this loss severely impairs NADPH oxidase activity. This provides a molecular explanation for the patient’s clinical presentation of recurrent severe infections. However, the precise effect of this mutation on protein function requires further validation through *in vivo* and *in vitro* experiments. Rae et al. (11)clearly demonstrated that when *CYBA* gene mutations occur in the transmembrane domain encoding the p22-phox protein, patients exhibit the A22^(0) phenotype^(complete loss of protein expression). Mutations in non-transmembrane regions typically result in the A22^+^ phenotype (normal protein expression but loss of function). Rae et al. (11)systematically analyzed 9 new families with *CYBA* mutations and further clarified this genotype-phenotype correlation via *in vitro* cellular functional assays, including immunoblot detection of p22phox/gp91phox protein expression and reduced-minus-oxidized difference spectroscopy for flavocytochrome b558 content, though *in vivo* animal model validation for the direct mutation-phenotype causality was still lacking. The c.427C>T variant in our patient is located in the C-terminal proline-rich non-transmembrane functional region of p22phox, which is consistent with the mutation location reported in Rae et al. ’s or study for the A22^+^. However, unlike the missense mutations reported by Rae et al, our variant is a nonsense mutation causing premature translational termination at amino acid 143, which results in a truncated protein lacking the critical C-terminal proline-rich domain essential for p47phox binding, leading to complete loss of NADPH oxidase function. Combined with the patient’s completely abnormal NBT reduction and DHR respiratory burst assay results, this variant is inferred to result in the A22° phenotype (complete loss of functional p22phox protein). This discrepancy may be attributed to the different mutation types: Rae et al. mainly reported missense mutations in the C-terminal domain that only cause single amino acid substitutions, while our nonsense mutation leads to structural destruction of the entire C-terminal functional region, resulting in more severe structural and functional impairment of p22phox.

Compared to previously reported *CYBA* mutation cases, this patient exhibits unique clinical features. Kang (22) described 15 children with *CYBA*-mutated CGD, where 87% presented before age 1 with severe infections and poor prognosis. Badalzadeh (23) reported an average age of onset at 7 months in 22 patients, with 31.8% experiencing BCG disease. Nearly 82% of patients had consanguineous parents, consistent with the present case. In contrast, the present patient presented at 4 years and 9 months (preschool age) with predominantly pulmonary infections without involvement of other organs like the liver or spleen, resulting in a relatively milder disease course. This difference may correlate with the mutation site location: the mutation in this case occurred at amino acid position 143, resulting in a truncated protein that retained partial N-terminal functional domains. In contrast, previously reported early-onset severe cases predominantly involved N-terminal truncation mutations (e.g., p.Q58*) leading to complete loss of protein function (14). This finding suggests that different *CYBA* mutation sites may lead to clinical phenotype heterogeneity, providing new evidence for genotype-phenotype correlation studies in CGD.

Therapeutically, hematopoietic stem cell transplantation (HSCT) remains the only curative treatment for CGD, restoring normal phagocytic function and enabling long-term disease-free survival (24). Multicenter studies report overall survival rates of 91.4% and event-free survival rates of 81.4% following HSCT in pediatric CGD patients (25). This patient remained infection-free for one year post-HSCT, validating the efficacy of HSCT in treating AR-CGD. Furthermore, the core of long-term CGD management involves infection prophylaxis. Standardized use of co-trimoxazole (for bacterial and Pneumocystis jirovecii infections) and itraconazole (for fungal infections) significantly reduces infection frequency (26). In this case, the patient initially received co-trimoxazole for bacterial infection prevention and itraconazole for fungal infection prevention, combined with anti-tuberculosis therapy to control disseminated BCG disease, effectively reducing infection frequency.

A limitation of this study is that while bioinformatics predicted the c.427C>T mutation causes protein truncation and loss of function, we lack *in vitro* or cellular functional assays (e.g., Western blot detection of p22phox protein expression) to further validate the mutation’s impact on protein function. Such functional assays represent the gold standard for confirming pathogenicity and constitute a future research direction. Nevertheless, based on rigorous ACMG guideline-based grading, a characteristic clinical phenotype, and parental validation, we maintain that this variant is the causative agent in this patient.

In summary, early immunological evaluation and genetic testing should be initiated for children with unexplained recurrent severe infections to determine whether they have a rare primary immunodeficiency disorder. The novel *CYBA* gene variant c.427C>T (p.Q143*) identified in this study not only expands the spectrum of pathogenic variants in this gene but also provides important reference for the clinical diagnosis and genetic counseling of CGD.

## Data Availability

The raw data supporting the conclusions of this article will be made available by the authors, without undue reservation.
